# Seaweed assemblages under a climate change scenario: Functional responses to temperature of eight intertidal seaweeds match recent abundance shifts

**DOI:** 10.1038/s41598-018-31357-x

**Published:** 2018-08-28

**Authors:** Cristina Piñeiro-Corbeira, Rodolfo Barreiro, Javier Cremades, Francisco Arenas

**Affiliations:** 1 BioCost Research Group, Facultad de Ciencias and Centro de Investigaciones Científicas Avanzadas (CICA), Universidad de A Coruña, 15071 A Coruña, Spain; 2 CIIMAR, Centro Interdisciplinar de Investigação Marinha e Ambiental, Terminal de Cruzeiros do Porto de Leixões, Av. General Norton de Matos s/n, 4450-208 Matosinhos, Portugal

## Abstract

Field evidence is essential to assess the consequences of climate change but a solid causal link often requires additional information obtained under controlled laboratory conditions. Additionally, the functional response to temperature may also help to discriminate species potentially more vulnerable to warming. Using a highly resolved temperature gradient, we examined the temperature dependence of photosynthesis and respiration in eight intertidal seaweeds that recently followed opposite abundance trends in NW Iberia. The temperature dependence of photosynthesis was consistently different between the macroalgae that increased and those that decreased their abundance in the last decade and a half, with photosynthesis twice more sensitive in the upward group. Unlike photosynthesis, the temperature dependence of respiration was unrelated to the abundance trend group, implying that the net metabolic scaling with temperature varied between the two groups of seaweeds. Overall, our results provide experimental support to the role of temperate as a likely driver of the changes in abundance recorded by field-monitoring studies. They also suggest that the temperature dependence of photosynthesis and respiration assessed in short-term experiments may serve as a biomarker of the potential vulnerability of some seaweed to the consequences of water warming.

## Introduction

Macroalgae are the dominant primary producers in coastal areas, providing essential ecosystem functions and services such as habitat and/or food for many marine organisms^1^. Thus, shifts in the composition of seaweed assemblages can have large consequences for the entire coastal community^2,3^. Temperature is widely acknowledged as a key determinant of the geographic range of marine benthic macroalgae and the large-scale biogeographic distribution pattern of many seaweeds has been explained by coupling species’ thermal traits with local seawater temperatures^1,4^.

Seawater temperatures are changing worldwide and the global oceans warmed an average of 0.1 °C per decade over the last 40 years^5^. Although a global phenomenon, the warming rate varies among regions/latitudes^6^. For example, sea surface temperature (SST) near the coast along the Iberian Peninsula seems to have warmed somewhat faster than the global average^7^. Understandably, the consequences of ocean warming for marine ecosystems have received increasing attention in recent years. There is a growing evidence that increasing temperatures have direct physical consequences for marine organisms and that warming, jointly with other anthropogenic stressors, is already affecting marine communities^3,8–10^.

In the particular case of the seaweeds, range shifts and abundance changes have been documented for various regions around the world^10–14^. In the Northeast Atlantic, some northern cold-adapted canopy forming seaweeds decreased in abundance in recent years while other meridional species with an affinity for warmer temperatures increased in regions such as North Spain, French Brittany, or British Isles^10,11,13,15,16^. Further North, warming seems responsible for the increased kelp biomass and the shift to a shallower depth of the biomass peak recently reported for one kelp forest at the border of the Artic Ocean^17^. Elsewhere, a rapid climate-driven regime shift has been reported for an extensive portion of the Australian temperate reef communities, where mixed kelps-fucacean canopies were lost after a succession of extreme heat waves and were replaced by persistent seaweed turfs with consequences for fish and marine invertebrate assemblages^14^. Previously, large-scale destructions of kelp forests were observed along the Pacific coasts of America after extreme climatic events but, unlike Australia, these forests recovered as environmental conditions returned to normal^18,19^

Range shifts and/or abundance changes are essential evidences for a realistic assessment of the consequences of rising temperatures for macroalgae^3^. However, field-monitoring studies may still be insufficient for establishing causal relationships or to foresee the effects of an even warmer future. Our inference ability can be greatly improved by coupling field observations with analyses of the functional response of seaweeds to increasing temperature under controlled laboratory conditions^20–23^. In thermo-conformer organisms like seaweeds, ambient temperature influences biochemical reactions with direct consequences for key physiological rates such as photosynthesis and respiration^24^. Respiration generally increases with temperature without reaching a clear optimum, while photosynthesis typically rise up to a plateau at a maximal level and then rapidly declines near the upper critical temperature^1,25^. Temperature optima for photosynthesis tend to be in agreement with the distribution range and species from colder latitudes often reach peak values at lower temperatures than temperate or tropical ones^4,25–28^.

Another thermal trait that also varies among species and even among populations, is the temperature dependence of metabolic rates^1^. The latter is typically characterized with Q_10_, the factor by which a rate increases for a 10 °C rise in temperature. However, parameterizing in 10 °C increments introduces a distortion and the Boltzmann-Arrhenius model derived from chemical kinetics is a better alternative to characterize the temperature dependence^29,30^. In this formulation, the temperature dependence is given by the slope of the Boltzmann-Arrhenius model, which with sign reversed is known as the activation energy *E*_*a*_^31^. Unlike Q_10_, *E*_*a*_ is temperature independent and avoids the distortion introduced by parameterizing in 10 °C increments^29,30^. Thermal dependence in seaweeds can be greatly affected by ambient temperature and metabolic rates often increase more in response to increasing temperature when the individuals grew in cooler environments^32,33^. As a result, a higher temperature dependence has been reported for individuals collected in winter compared to those sampled in summer^34^, or in populations from cooler locations along a latitudinal gradient^35^. Individuals from cooler environments also experienced faster drops in net photosynthesis when they were exposed to temperatures that surpassed their temperature optimum^35^.

Despite the growing number of reports on the impact of global change on the distribution and abundance of seaweeds, few studies have compared the thermal functional response of seaweeds that followed divergent abundance trends in recent years^22,36^. This study examines the physiological response to an experimental temperature gradient in eight intertidal seaweeds that changed their patterns of site occupancy differently in NW Iberia in the last decades: four that decreased their frequency and four that increased^10^. To provide further support for the role of temperature as a decisive driver of the observed shifts, we tested whether there were consistent differences in the functional responses to temperature shown by each set of seaweeds. The seaweeds targeted in this study are major members of the seaweed assemblage in NW Iberia^37,38^; a better knowledge of the actual drivers of their recent frequency shifts seems highly relevant to predict the future configuration of the intertidal ecosystem.

## Material and Methods

### Sample collection

*Mastocarpus stellatus* (Stackhouse) Guiry*, Chondrus crispus* Stackhouse*, Himanthalia elongata* (Linnaeus) S.F.Gray and *Fucus vesiculosus* Linnaeus were chosen as representatives of seaweeds with a downward trend in site occupancy. A previous survey showed that these species had 48% (*M. stellatus*) to 70% (*C. crispus, H. elongata, F. vesiculosus*) lower site occupancy in 2014 than in 1998/99^10^. On the other hand, *Corallina caespitosa* R.H.Walker, J.Brodie & L.M.Irvine*, Bifurcaria bifurcata* R. Ross*, Cystoseira tamariscifolia* (Hudson) Papenfuss and *Cystoseira baccata* (S.G.Gmelin) P.C.Silva were selected as representatives of seaweeds that followed an upward trend because they experienced gains of 11% (*C. tamariscifolia*) to 75% (*C. caespitosa*) in the frequency of occupied sites over the same period of time^10^.

Live fronds of the eight macroalgal species were collected from intertidal locations in NW Iberia in February 2016. The study included Fucales with apical growth (*H. elongata, F. vesiculosus, C. baccata, C. tamariscifolia, B. bifurcata*) and multiaxial red algae with pseudoparenchymatous tissues where the growth of new tissue is not restricted to a meristematic region (*M. stellatus, C. crispus, C. caespitosa*). To guarantee that the functional responses were consistently recorded in metabolically active tissue, samples consisted of whole fronds in the case of the pseudoparenchymatous red algae and the moderately sized *B. bifurcata*. By contrast, the samples of large-sized Fucales consisted of moderately sized young plants (*F. vesiculosus*), plants with young receptacles (*H. elongata*), or distal, young apical fronds (*C. tamariscifolia* and *C. baccata*) were used for convenience (frond size 15 cm long in all cases). As a result, whole fronds samples and young frond/tissue regions were evenly partitioned between the two trend sets compared in our experimental design. Samples were transported in a portable cool box to the laboratory where grazers and epiphytic organism were carefully removed. For each species, a subset of evenly sized specimens was acclimated to laboratory conditions before the experiment during two weeks with aerated sea water at around 13 °C; water was renewed every two days along the acclimation period to avoid nutrient depletion.

### Functional responses: estimation of productivity and respiration rates

To examine the functional response of the target species to rising temperatures, we assessed the respiration and primary productivity rates over a thermal gradient under controlled laboratory conditions. Respiration and net primary productivity were determined throughout oxygen fluxes by continuously recording (every 30 seconds) the concentration of dissolved O_2_ with a HQ40D oxygen probe (Hach Lange^®^) within incubation chambers. Seaweed individuals were incubated under temperature-controlled conditions in custom-made, sealed methacrylate incubation units (2 L) equipped with a submersible pump to homogenize the medium. Lighting was provided by a variable number of 30 W Biolux Osram^®^ fluorescent lamps. The setup allowed the simultaneous and independent incubation of three samples, successively exposed for 20 min (30 min for dark conditions) to seven light intensities (0, 30,108, 237, 557, 971, 1515 µmol photons · m^−2^ · s^−1^). Light intensity ranged from light-limited conditions (dark incubation) to light-saturation and allowed us to calculate photosynthesis-irradiance (P-E) curves. Independent P-E curves were constructed at nine temperatures for each species. The experiment included water temperatures normally experienced by seaweeds in NW Iberian along the year (12 °C, 15 °C, 18 °C and 21 °C), but also extremes beyond the limits usually seen in the region (6 °C, 9 °C, 24 °C, 27 °C and 30 °C)^39,40^. Samples were acclimated to incubation-temperature for 12 h before each experiment. P-E curves for each combination of species and temperature were recorded following a strictly randomized order to avoid any bias. In addition, filtered sea water (5 µm) was used to prevent any spurious influence of phytoplankton on photosynthesis/respiration estimates. The effectiveness of the filtering procedure was repeatedly confirmed along the experiment by recording O_2_ changes in control (no seaweed) incubation units. After incubation, samples were oven-dried at 45 °C for 48 h to estimate dry biomass. Dark respiration (*R*_d_) and net productivity (*NP*) rates were estimated as the slope of the linear regression between O_2_ concentration and time; gross photosynthesis (*GP*) was calculated by adding the absolute value of *R*_d_ to estimates of *NP* at each light intensity. All rates were expressed as mg O_2_ · g^−1^ DW · h^−1^.

The maximum rate of gross photosynthesis at saturating light (*GP*_max_) and the photosynthetic efficiency at low irradiance (α) were determined by fitting the model of Eilers and Peeters^41^:

1 $$GP=1/({E}^{2}/\alpha \times {E}_{opt}^{2})+(E/G{P}_{max})-(2E/\alpha \times {E}_{opt})+(1/\alpha )$$

where *E*_opt_ is the optimum light intensity. The maximum rate of net photosynthesis (*NP*_max_) was calculated as *GP*_max_ − *R*_d_. Model fitting was done by non-linear regression with the help of the package Phytotools^42^ for R^43^.

*T*_opt_ and rate at *T*_opt_ of *NP*_max_, *GP*_max_ and *R*_d_ were estimated by fitting the three parameter Gaussian model recommended by June, *et al*.^44^:

2 $$y={y}_{opt}{e}^{(-0.5{((T-{T}_{opt})/b)}^{2})}$$

where *y* is the rate (*NP*_max_, *GP*_max_, or *R*_d_) estimated at each temperature *T*, *y*_opt_ is the value of the rate at the optimum temperature (*T*_opt_) and *b* is the steepness of the curve. The Gaussian model was fitted with the non-linear routine implemented in Statgraphics Centurion XVI (StatPoint Technologies, Inc.).

### Activation Energy from the Arrhenius equation

The temperature dependence of photosynthesis and dark respiration was described with the Boltzmann-Arrhenius model:

3 $$R={R}_{0}{e}^{-{E}_{a}/kT}$$

where *R*_0_ is a scaling coefficient and $\,{e}^{-{E}_{a}/kT}$ is the Boltzmann factor that describes an exponential relationship between rate and temperature (*T* in kelvin). In this model, the temperature dependence of the trait is measured by the activation energy *E*_*a*_, while *k* is the Boltzmann’s constant^31^. Accordingly, we calculated the *E*_*a*_ of *GP*_max_, *NP*_max_ and *R*_d_ for each species. Since the Arrhenius model describes an exponential relationship while the temperature response function can be hump-shaped, we fitted the model only to the approximately exponential rise phase of our rate-temperature curves^45^. The rising component of each curve was delimited by iteratively removing rate measurements at the upper terminal temperatures until monotonicity was observed in the response. To do this, a quadratic function was initially fitted to the relationship between the log-transformed rate and 1/*kT* using ordinary least squares (OLS) regression. If the *p*-value of the model coefficient for the order 2 term was significant, the response was considered hump-shaped, the highest terminal temperature removed and the procedure repeated until the model coefficient for the order 2 term was non-significant. Then, a linear model was fitted to the data and we used R^2^-adjusted values to assess whether the linear model fitted better than the quadratic one; if necessary, the highest terminal temperature was removed until the R^2^-adjusted value was equal or larger for the linear than for the quadratic model. The whole procedure was accomplished with the help of Statgraphics Centurion XVI (StatPoint Technologies, Inc.).

### Data analysis

For each physiological rate (*GP*_max_, *NP*_max_, *R*_d_ and α), we used conditional sums of squares as a global test to assess whether the activation energy differed among species^46^. Then, we calculated 95% confidence intervals corrected for multiple comparisons with the Bonferroni method to determine species or sets of species with significantly different activation energies. Bonferroni-adjusted 95% confidence intervals were likewise used to assess interspecific differences in *T*_opt_ and in rate estimates at *T*_opt_.

## Results

### Effects of temperature on photosynthesis and respiration

**Figure 1:**
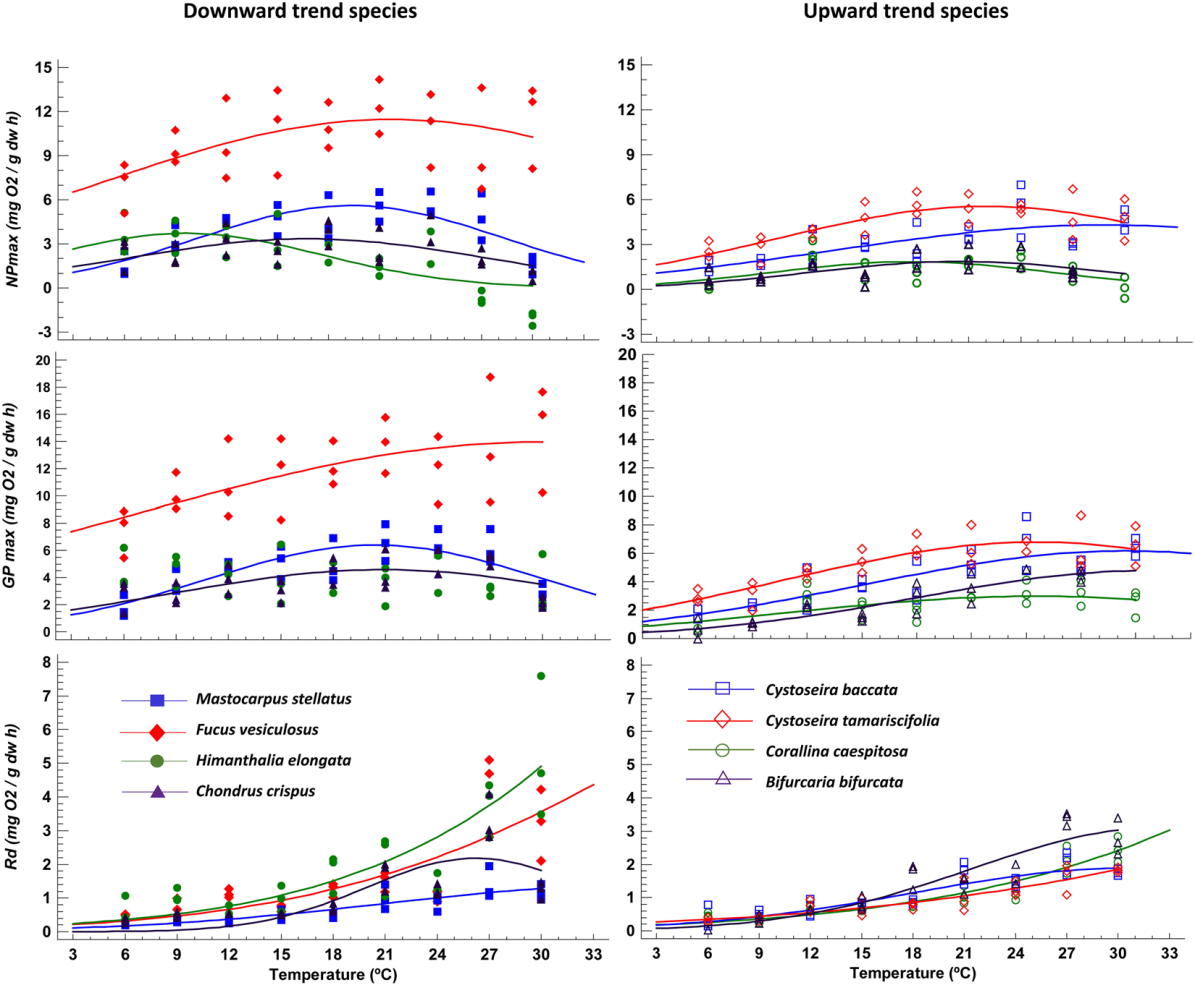
Temperature dependence of *NP*_max_ (top), *GP*_max_ (middle) and *R*_d_ (bottom) in seaweeds that showed divergent downward (left) and upward (right) abundance trends in NW Iberia along the last decade. Lines are non-linear fits of a three-parameter Gaussian model (see text for further detail). Due to poor fitting, no line is shown for *GP*_max_ in *H. elongata*.

Irrespective of temperature, both net and gross maximum photosynthesis were conspicuously higher in *F. vesiculosus* than in other seaweed species. Metabolic estimates in *F. vesiculosus* ranged 5.1–14.2 mg O_2_ · g^−1^DW · h^−1^ for *NP*_max_ and 5.5–18.7 mg O_2_ · g^−1^DW · h^−1^ for *GP*_max_ compared to 2.6–7.0 mg O_2_ · g^−1^DW · h^−1^ for *NP*_max_ and 0.0–8.7 mg O_2_ · g^−1^DW · h^−1^ for *GP*_max_ in other algae (Fig. 1). Other than *F. vesiculosus*, the productivity rates were very similar in downward and upward seaweeds. Regardless of the species, the response of *NP*_max_ and *GP*_max_ to temperature was consistently unimodal; photosynthesis increased gradually with temperature up to an optimum and decreased afterwards. Nonetheless and despite the consistently unimodal shape, there were differences between species. For example, the fall phase at temperatures beyond the optimum seemed more pronounced in three of the four downward seaweeds (not in *F. vesiculosus*) than in upward algae. No differences were found in the photosynthetic efficiency at low irradiance (α) between upward and downward trend species (results not shown).

**Figure 2:**
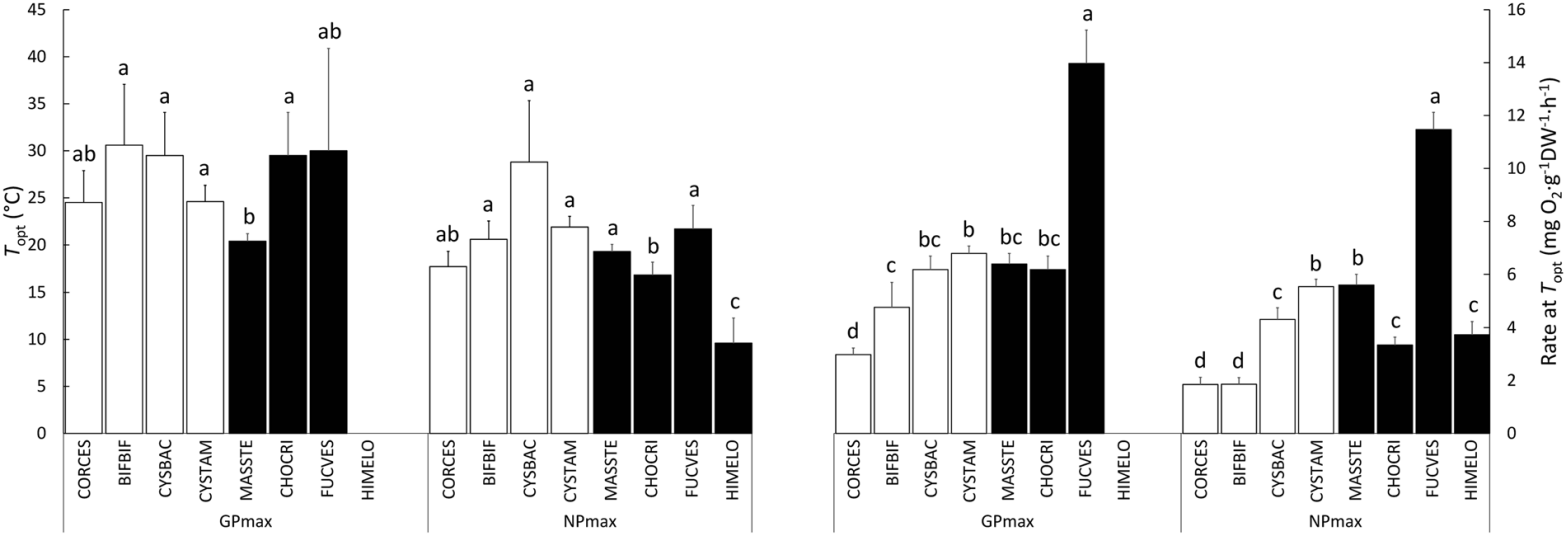
Comparison of *T*_opt_ and rate at *T*_opt_ for *GP*_max_ and *NP*_max_ estimated by non-linear regression for eight seaweeds that followed divergent upward (white bars) and downward (black bars) abundance trends in NW Iberia along the last decade. 95% confidence intervals calculated after adjusting the experiment wise error rate with the Bonferroni method. No bar shown for *GP*_max_ in *H. elongata* due to poor fitting. See Table 1 for species abbreviations.

Gaussian model estimates of *T*_opt_ differed significantly among species both for *GP*_max_ and for *NP*_max_ (Table 1 and Fig. 2). However, no consistent pattern could be detected between the upward and downward sets of species. Instead, many *T*_opt_ estimates for *NP*_max_ ranged between 17 °C and 22 °C and were statistically undistinguishable. Exceptions were the downward *H. elongata* (9.6 ± 2.67 °C) and the upward *C. baccata* (28.8 ± 6.54 °C). *T*_opt_ estimates for *GP*_max_ were slightly higher than for *NP*_max_, ranging between 20 °C and 31 °C. No estimate could be obtained for *H. elongata*, but an examination of the original data reveals that the largest rates of *GP*_max_ also occurred at low temperatures around 6–9 °C. Like *T*_opt_, Gaussian model estimates of *NP*_max_ and *GP*_max_ at *T*_opt_ also varied significantly among species. Again, the highest and the lowest estimates fell into algae from different trend sets: the downward *F. vesiculosus* (11.5 ± 0.65 mg O_2_ · g^−1^DW · h^−1^ *NP*_max_ and 14.0 ± 1.26 mg O_2_ · g^−1^DW · h^−1^ *GP*_max_) and the upward *C. caespitosa* (1.9 ± 0.27 mg O_2_ · g^−1^DW · h^−1^ *NP*_max_ and 3.0 ± 0.25 mg O_2_ · g^−1^DW · h^−1^ *GP*_max_). Still, no consistent pattern could be detected between abundance trend groups for any of these parameters.

**Table 1:** Parameter estimates from fitted temperature-responses curves for *GP*_max_, *NP*_max_ and *R*_d_ of macroalgae that decreased (“downward”) or increased (“upward”) their frequency of occurrence in NW Iberia in recent times.

Occurrence trend	Species^a^	*GP*_max_		*R*_d_		*NP*_max_	
		*T*_opt_	Rate at *T*_opt_	*T*_opt_	Rate at *T*_opt_	*T*_opt_	Rate at *T*_opt_
Upward	CORCES	24.5 ± 3.39	3.0 ± 0.25	ND	2.4 ± 0.22	17.7 ± 1.62	1.9 ± 0.27
Upward	BIFBIF	30.6 ± 6.47	4.8 ± 0.94	ND	2.8 ± 0.32	20.56 ± 1.94	1.9 ± 0.25
Upward	CYSBAC	29.5 ± 4.58	6.2 ± 0.51	ND	1.8 ± 0.06	28.8 ± 6.54	4.3 ± 0.44
Upward	CYSTAM	24.6 ± 1.73	6.8 ± 0.28	ND	1.8 ± 0.05	21.9 ± 1.13	5.5 ± 0.28
Downward	MASSTE	20.4 ± 0.80	6.4 ± 0.40	ND	1.2 ± 0.13	19.3 ± 0.76	5.6 ± 0.39
Downward	CHOCRI	29.5 ± 4.58	6.2 ± 0.51	26.2 ± 1.34	2.2 ± 0.25	16.8 ± 1.37	3.4 ± 0.33
Downward	FUCVES	30.0 ± 10.89	14.0 ± 1.26	ND	3.2 ± 0.61	21.7 ± 2.49	11.5 ± 0.65
Downward	HIMELO	ND	ND	ND	5.3 ± 1.22	9.6 ± 2.67	3.7 ± 0.50

*T*_opt_ (°C) is the temperature at which maximum rates occur; rates at *T*_opt_ were determined for *GP*_max_ and *N*P_max_ (mg O_2_ · g^−1^DW · h^−1^). For most species, *T*_opt_ could not be determined for R_d_ (ND = not determined); in those cases, *R*_d_ at *T*_opt_ is the rate recorded at 30 °C. Values are estimate ± asymptotic SE except for *R*_d_ at *T*_opt_ where values a mean ± SE.

^a^CORCES = *Corallina caespitosa*; BIFBIF = *Bifurcaria bifurcata*; CYSBAC = *Cystoseira baccata*; CYSTAM = *Cystoseira tamariscifolia*; MASSTE = *Mastocarpus stellatus*; CHOCRI = *Chondrus crispus*; FUCVES = *Fucus vesiculosus*; HIMELO = *Himanthalia elongata*.

Unlike photosynthesis, no *T*_opt_ could be observed for dark respiration (*R*_d_) because it increased monotonically with temperature in most algae (Fig. 1). The exception was the downward Irish moss *C. crispus* where *R*_d_ slightly decreased at the highest temperature, resulting in a *T*_opt_ estimate of 26.2 ± 1.34 °C (*R*_d_ estimate at a *T*_opt_ = 2.2 ± 0.25 mg O_2_ · g^−1^DW · h^−1^). In most cases, *R*_d_ changed gradually with temperature, although some downward seaweeds (*H. elongata* and *F. vesiculosus*) showed some evidence of an exponential increment of *R*_d_ at the highest experimental temperatures (>24 °C).

### Activation energy

**Figure 3:**
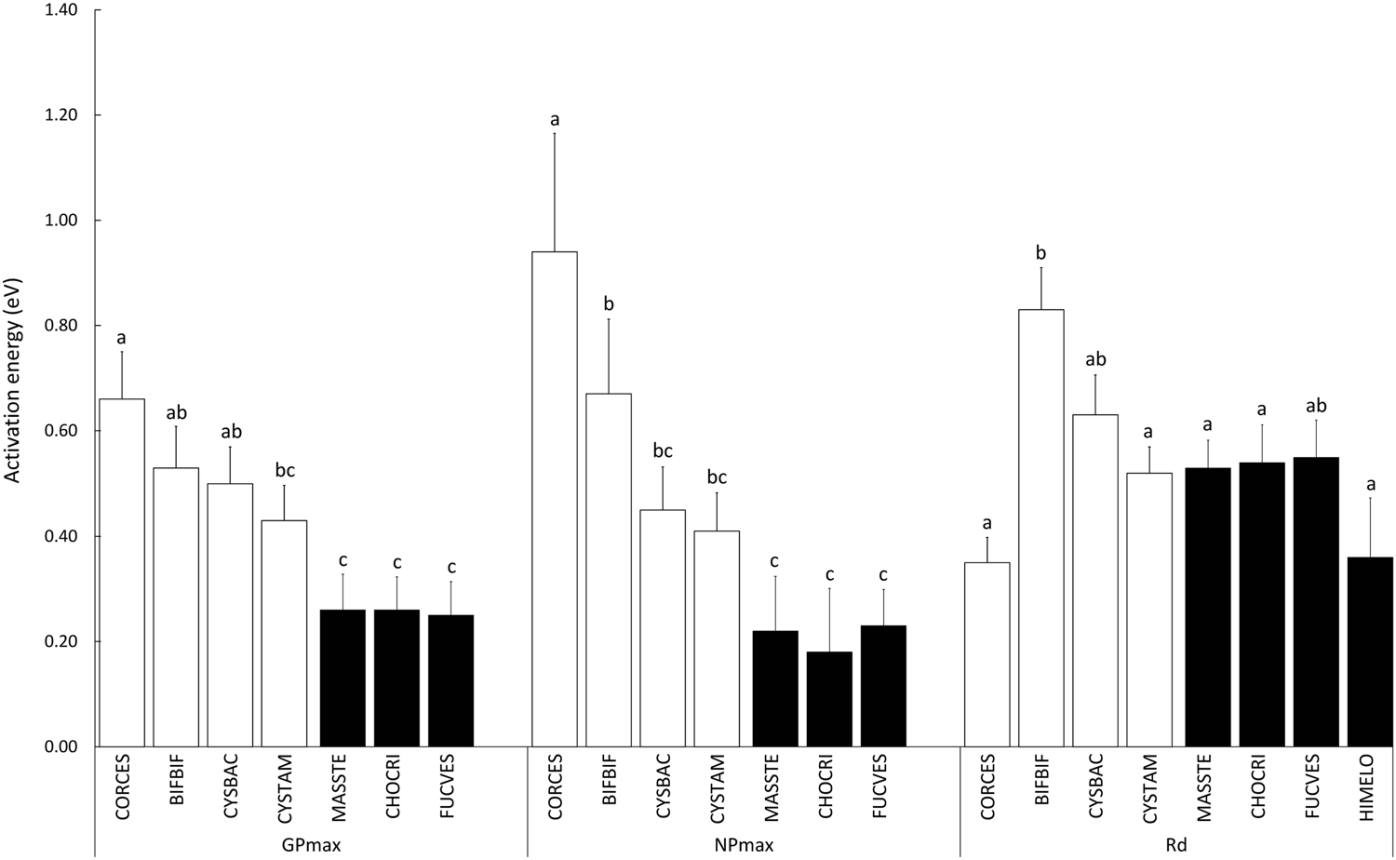
Comparison of *E*_a_ estimates for the rise responses of physiological rates *GP*_max_, *NP*_max_ and *R*_d_ for eight seaweeds that followed divergent upward (white bars) and downward (black bars) abundance trends in NW Iberia along the last decade. 95% confidence intervals calculated after adjusting the experiment wise error rate with the Bonferroni method. No bar shown for *GP*_max_ and *NP*_max_ of *H. elongata* due to poor fitting. See Table 1 for species abbreviations.

We were able to estimate activation energies for net and gross photosynthesis in seven of the eight seaweeds (Table 2). In the downward *Himanthalia elongata*, the range of experimental temperatures at which photosynthesis followed a monotonic increase was too small to allow a reliable estimate of *E*_*a*_. Unlike *T*_opt_, our analyses of the temperature dependence of photosynthesis with the Boltzmann-Arrhenius model suggested consistent differences between upward and downward algae. The conditional sums of squares of *E*_*a*_ estimates revealed highly significant interspecific differences in the temperature dependency of both *GP*_max_ (*F* = 4.4; *P*-value = 0.0004) and *NP*_max_ (*F* = 8.4; *P*-value < 0.0001). Upward species showed consistently higher activation energies than downward ones, indicating that productivity was more responsive to temperature increases below *T*_opt_ in the former. *E*_*a*_ ranged 0.43–0.66 eV for *GP*_max_ in upward algae compared to 0.25–0.26 eV in downward ones and Bonferroni-corrected pairwise comparisons indicated that the differences between both sets of species were statistically significant in most cases (Fig. 3). A similar pattern was observed for *NP*_max_. Again, net photosynthesis was more responsive to temperature in upward than in downward algae, with activation energies ranging 0.40–0.94 eV in the former while only 0.18–0.22 eV in the latter. Bonferroni-corrected pairwise tests again revealed significant differences between upward and downward algae, although the slightly wider confidence intervals of some estimates lowered the statistical power of some comparisons compared to *GP*_max_.

**Table 2:** Temperature dependence of *GP*_max_, *NP*_max_ and *R*_d_.

Occurrence trend	Species^a^	*GP*_max_				*R*_d_				*NP*_max_			
		*E*_*a*_	*R*^2^*-*adj	*P*-value	Range (°C)/*N*	*E*_*a*_	*R*^2^*-*adj	*P*-value	Range (°C)/*N*	*E*_*a*_	*R*^*2*^*-*adj	*P*-value	Range (°C)/*N*
Upward	CORCES	0.66 ± 0.090	80.3	<0.0001	6–24/21	0.35 ± 0.048	77.2	<0.0001	6–24/21	0.94 ± 0.225	65.9	0.0024	6–21/18
Upward	BIFBIF	0.53 ± 0.078	65.9	<0.0001	6–30/27	0.83 ± 0.080	86.4	<0.0001	9–27/21	0.67 ± 0.142	65.5	0.0005	6–21/18
Upward	CYSBAC	0.50 ± 0.070	74.0	<0.0001	6–24/21	0.63 ± 0.076	76.1	<0.0001	6–27/24	0.45 ± 0.082	62.2	<0.0001	6–24/21
Upward	CYSTAM	0.43 ± 0.067	71.8	<0.0001	6–21/18	0.52 ± 0.050	81.2	<0.0001	6–30/27	0.41 ± 0.073	66.8	<0.0001	6–21/18
Downward	MASSTE	0.26 ± 0.068	47.3	0.0016	9–24/18	0.53 ± 0.052	81.0	<0.0001	6–30/27	0.22 ± 0.069	40.1	0.0048	9–24/18
Downward	CHOCRI	0.26 ± 0.063	42.0	0.0004	6–27/24	0.54 ± 0.071	75.3	<0.0001	6–24/21	0.18 ± 0.104	14.0	0.1044	6–24/21
Downward	FUCVES	0.25 ± 0.064	49.4	0.0011	6–21/18	0.55 ± 0.070	71.8	<0.0001	6–30/27	0.23 ± 0.066	43.7	0.0028	6–21/18
Downward	HIMELO	−0.12 ± 0.067	9.0	0.0717	6–30/27	0.36 ± 0.113	35.8	0.0053	6–24/21	−0.30 ± 0.121	27.1	0.0223	6–30/27

Values are the activation energy *E*_a_ (±SE; eV) calculated from the Boltzmann-Arrhenius model by OLS fit of the rise component of the rate-temperature curve. Range (°C) is the range of temperatures where the rate followed a monotonic rise while *N* is sample size. R^2^-adjusted is the percentage of the variability explained by the fitted model while *P*-value indicates the significance of the *E*_a_ estimate.

^a^CORCES =* Corallina caespitosa*; BIFBIF = *Bifurcaria bifurcata*; CYSBAC = *Cystoseira baccata*; CYSTAM = *Cystoseira tamariscifolia*; MASSTE = *Mastocarpus stellatus*; CHOCRI = *Chondrus crispus*; FUCVES = *Fucus vesiculosus*; HIMELO = *Himanthalia elongata*.

A very different picture emerged when we analysed the temperature dependence of dark respiration (Table 2). The conditional sums of squares test detected statistically significant differences among seaweeds (*F* = 2.7; *P*-value = 0.0124). Nonetheless, the Bonferroni-corrected pairwise comparisons indicated that this was mostly a consequence of the extreme temperature dependence shown by respiration in the upward *B. bifurcata* (0.83 ± 0.080 eV) (Fig. 3). Many algae, both upward and downward, had activation energies for *R*_d_ around 0.52–0.63 eV and while respiration still seemed less dependent on temperature in some species, these included both upward (*C. caespitosa*) and downward (*H. elongata*) examples.

## Discussion

Growing experimental evidence indicates that losing species can diminish ecosystems functions such as productivity, biomass accumulation or nutrient uptake. However, the ecosystem consequences of species loss will probably be species-specific as functional performance varies among species^47^. In this context, finding simple proxies to anticipate a species’ susceptibility to become extinct will improve our ability to predict the actual consequences of future reductions in species diversity. Our results found that, besides the varying metabolic rates and optimal temperatures recorded in the species examined, the temperature dependence of photosynthesis (as the activation energy *E*_*a*_ for *GPP*) was consistently larger in seaweeds that recently followed an upward abundance trend than in those that declined in abundance in recent years. These variation is in agreement with the hypothesis that temperature may have played a pivotal role in the shifts in abundance detected in these species. Furthermore, they also suggest that *E*_*a*_ might be a convenient tool to foresee the impact of ocean warming on seaweed populations.

The metabolic theory of ecology (MTE) predicts that *E*_*a*_ for heterotrophic metabolism should be close to 0.65 eV (Brown *et al*. 2004) while photosynthetic *E*_*a*_ should be smaller, estimated around 0.32 eV by Allen, *et al*.^48^. However, these values are intended to characterize processes at a global scale and have been derived from approximations based on the kinetics of the relevant rate-limiting biochemical reactions, e.g. the approximation of 0.32 eV for the temperature dependence of photosynthesis assumes that the rate of C_3_ photosynthesis is limited by Rubisco carboxylation^48^. Consequently, the values predicted by MTE are found in interspecific studies^30,31^ or when a sizeable number of intraspecific estimates are combined into a single mean value^45^. Meanwhile, *E*_*a*_ estimates for single traits obtained in individual intraspecific studies are usually very variable and typically range between 0.2 and 1.2 eV, consistent with the variation observed for metabolic reactions^30,45^. Even the universality of a single value to characterize the temperature dependence of primary production at very large (ecosystem) scale has been questioned^49^ and it has been proposed that *E*_*a*_ for primary production might be larger in aquatic than in terrestrial ecosystems^50^. In this context, the fact that our *E*_*a*_ estimates for photosynthesis do not match the theoretical value of 0.32 eV 0.44 for aquatic ecosystems according to^50^ is just another example of the complexity of the temperature dependence of photosynthesis and is probably related to differences in ecology between species^51^. Unlike photosynthesis, the lack of consistent differences for respiration *E*_*a*_ among species with upward and downward abundance trends implies that respiration responded to temperature more uniformly across species than production. Moreover, the average respiration *E*_*a*_ in our experiment was 0.54 ± 0.05 eV, below the 0.65 eV derived from theory but nearly identical to the median of 0.55 eV found in an comprehensive analysis of intraspecific responses to temperature^45^, indicating a better match with the predictions of MTE.

Temperature dependence is known to vary as a result of processes such as acclimation, acclimatization and adaptation^29^. In seaweeds, for example, sensitivity to increasing temperatures can be greater in winter than in summer, although this change appears to be more pronounced in polar than in temperate seaweeds^1,34^. Even within a single species, the temperature dependence of photosynthesis and respiration has been shown to gradually decrease from cooler to warmer latitudes^35^. In our case, because we used seaweeds collected at the same time of the year that were kept under uniform environmental conditions until the experiment, both acclimation and acclimatization can be safely disregarded as explanations for the differences in photosynthesis *E*_*a*_ detected between the two groups of species. Likewise, although our experimental design combined whole fronds for some species with young or distal apical fronds for others, the two types of sample were evenly represented in both trend groups, suggesting the sample type was not responsible for the consistent differences in *E*_a_ between trend groups. Instead, the variation in photosynthesis *E*_*a*_ possibly indicates that each group has a different potential to cope with the physiological consequences of water warming.

A comparison of the Boltzmann factor shows that the temperature dependence of respiration and photosynthesis was very similar in the upward trend group, predicting an average increase of twofold over the temperature range 283–293 K (10–20 °C) (${e}^{-{E}_{a}/293k}/{e}^{-{E}_{a}/283k}=2.33$ for *R*_d_, 2.11 for *GP*_max_ and 2.48 for *NP*_max_). In contrast, photosynthesis was much less sensitive to temperature (${e}^{-{E}_{a}/293k}/{e}^{-{E}_{a}/283k}=1.28$ for *GP*_max_, 1.17 for *NP*_max_) than respiration (2.01 for *R*_d_) in the downward trend, suggesting a higher risk to experience energy imbalances when the temperature increases over the physiological range. A comparable, but more extreme, imbalance between the temperature dependence of photosynthesis and respiration has been observed in Antarctic red algae^52^, suggesting that it might be characteristic of seaweeds adapted to low temperatures. In comparison, the temperature dependence of respiration is usually similar to or lower than that of photosynthesis in seaweeds from temperate and warm latitudes^53–56^ but see^23,57^.

None of the other metabolic traits examined by us showed consistent differences among the two groups of species. Instead, functional responses were species-specific for physiological traits such as *T*_opt_, maximum *GPP* and *NPP*, dark respiration at *T*_opt_, or photosynthetic efficiency at low irradiance (alpha). Idiosyncratic or species-specific photosynthetic and respiration rates were expected since the seaweed examined here are considerably separated from a phylogenetic point of view. Metabolism in seaweeds is affected not only by the physical environment but also by biotic factors such as morphology, ontogeny, circadian and seasonal rhythms^1^. Studies on intraspecific and interspecific differences in temperature-photosynthesis response curves for many seaweed species indicate that both the amplitude and the position of the curve are usually correlated with the local temperature regime, reflecting local temperature adaptation^25^. For example, Antarctic seaweed populations show maximum net primary productivity rates at relatively cold temperatures, i.e. between 0 and 15 °C^52^, while tropical species of the genus *Asparagopsis* show maximum photosynthesis rates at 26 °C^58^. Intraspecific differences among populations revealing local adaption within species have been also recorded in seaweeds like *Chondrus crispus*^33^ or the kelp *Ecklonia radiata*^55^.

Our proposal that the activation energy of photosynthesis and respiration may help to foresee the vulnerability to water warming does not necessarily imply that temperature alters the abundance of these seaweeds through an impact on the rates of photosynthesis and respiration. It has long been known that the geographic boundaries of seaweeds are set by limitations to growth (temperature inappropriate for population growth), but also by limitations to survival (temperature prevents the survival of the hardiest stage) or to reproduction (temperature prevents the maturation of some stage)^25,59^. Therefore, we suggest that the temperature dependence of photosynthesis and respiration might serve as a biomarker of the ability of some seaweeds to cope with water warming, even though temperature may actually have a stronger, more influential impact on life cycle traits other than respiration or production (e.g. reproduction).

Temperature is the main abiotic factor driving large scale distribution of seaweed species^4,60^. In the North Atlantic, large shifts in the distribution patterns of marine species have been experienced during glacial-interglacial cycles shaping the contemporary distribution and the genetic structure of seaweed populations^61,62^. In the last decades, quick shifts in species distribution^3,10–13,15^ have been linked with rising temperatures in our oceans. Our experiment was aimed to characterize functional traits which could be relevant to assess seaweeds vulnerability to ocean warming. Previous, experimental studies have successfully used empirical lethal thresholds to define the current and future distribution of seaweeds across large spatial scales^63,64^. Here we found consistent differences in the temperature dependence of photosynthesis among species with opposite abundance trends, suggesting divergences in the metabolic scaling with temperature among these groups of seaweeds. Overall, our study supports the idea that temperature could be mechanistic cause of the shifts in abundance observed for these seaweeds in the last decades and provides a relatively simple experimental approach that could be further examined in other seaweeds undergoing similar climate-linked abundance changes.
